# Central and mixed apneas in children with obstructive sleep apnea: effect of adenotonsillectomy

**DOI:** 10.1007/s00405-023-08442-7

**Published:** 2024-01-16

**Authors:** Joselina Antunes, João Carvalho, Carolina Marinho, Sofie Vanderpoorten, Cristina Adónis, Filipe Freire

**Affiliations:** Otorhinolaryngology, Head and Neck Surgery Department, Professor Doutor Fernando Fonseca Hospital, IC19, 2720-276 Amadora, Portugal

**Keywords:** Adenotonsillectomy, Central sleep apneas, Mixed sleep apneas, Obstructive sleep apnea

## Abstract

**Purpose:**

Investigate the effect of adenotonsillectomy on mixed apnea index (MAI) and central apnea index (CAI) in children with moderate-to-severe obstructive sleep apnea syndrome (OSAS).

**Methods:**

Observational retrospective analysis of polysomnographic data in children diagnosed with moderate-to-severe OSAS and without comorbidity, submitted to adenotonsillectomy.

**Results:**

Data were available for 80 children, 55 boys and 25 girls, with a median age of 3.6 years (2.1–5.9). Before surgery AHI was 14.1 (11.0–18.4) per hour, with a median preoperative OAI of 7.1 (4.1–10.6), MAI of 1.2 (0.6–1.6) and CAI of 1.0 (0.4–2.0). Adenotonsillectomy caused significant improvements in MAI, from 1.2 (0.6–1.6) to 0.5 (0.1–0.8) (*p* < 0.001) and CAI from 1.0 (0.4–2.0) to 0.5 (0.1–0.9) (*p* < 0.001). This represents a normalization of MAI in 91.7% and CAI in 75.6% of children that had an abnormal value prior surgery.

**Conclusion:**

Non obstructive apneas are common in children with OSAS. Adenotonsillectomy caused significant decrease not only in OAI, but also in MAI and CAI in children with moderate-to-severe OSAS.

## Introduction

Sleep apnea is the result of respiratory airflow pauses and has three major types: obstructive sleep apnea (OSA), mixed sleep apnea (MSA) and central sleep apnea (CSA) [1]. OSA is thought to occur in about 4–18% of healthy children, MSA in 0–6% and CSA in 1–37% [2–5]. All three types of apnea indices are higher in neonatal period, and tend to decrease with age [6]. Current data suggest that in children, the upper limit of normal values for obstructive and mixed apnea indices is less than one per hour [3, 6]. The minimum number of CSA required to cause a specific disorder remains elusive, but despite that, some authors consider a central apnea index (CAI) ≥ 1 to be abnormal [3, 7]. A finding of ≥ 5 central apneas per hour is more consensually considered clinically relevant, as it can be associated with underlying conditions such as anatomical brain and brainstem abnormalities, neurogenetic conditions, upper airway abnormalities, prematurity, gastroesophageal reflux, obesity, hypothyroidism and in the context of other sleep-disordered breathing (SDB) [3, 7, 8].

MSA have characteristics from both central and obstructive apneas. The cause of mixed apneas in patients with obstructive sleep apnea syndrome (OSAS) seems to be ventilatory control instability and upper airway collapsibility, as they increase along with OSAS severity [9]. The clinical implication of MSA has not been fully understood, and so, the impact of MSA events in OSAS tends to be ignored, and considered to be part of OSAS [1, 10]. Some studies show that patients with OSAS and also MSA tend to show a poorer compliance to continuous positive airway pressure (CPAP), and also a poorer respiratory control stability [10]. Central apneas are common in children with OSAS, and seems to be caused by arousals due to obstructive events. The variability of CAI in children with OSAS may be due to individual sensitivity of chemoreceptors to changes in partial pressure of carbon dioxide [7, 8].

American Academies of Otorhinolaryngology, Pediatrics and Sleep Medicine agree that adenotonsillectomy (AT) should be considered the first-line treatment for children with OSAS. AT causes a significant improvement in SDB in the majority of otherwise healthy, non-obese children, with a success rate of approximately 75%. Despite that, it is defended that some groups of children with predominantly nonobstructive events may benefit from other management options, as tonsillectomy may not be indicated [11, 12]. More recent studies show that when severe CSA appears associated with OSAS, it is likely to resolve following AT, even in cases of children with neurologic abnormalities. More authors defend the treatment of OSAS with AT prior to additional workup to identify etiology of CSA in children with persistent central events [3, 7, 8].

This study aims to show the effect of AT on apnea events, namely on central and mixed, in children diagnosed with OSAS. We theorize that AT may improve not only obstructive events, but also mixed and central events.

## Methods

This was a retrospective study, performed at a single medical center (Professor Doutor Fernando Fonseca Hospital, Lisbon) between 2010 and 2020. The study was approved by local health ethics committee, and it was designed and conducted in compliance with the principles of Good Clinical Practice regulations and the Helsinki declaration.

Since 2010, polysomnographic data from children with an Apnea Hypopnea Index (AHI) ≥ 10 per hour were recorded in a database system for evaluation of surgical success. Patient data were analyzed for possible inclusion in this study. Patients were considered for inclusion in this study if they were submitted to AT to treat OSAS and apart from the preoperative polysomnography (PSG) were also submitted to a postoperative PSG, 3–6 months following surgery. Patients with one or more of the following conditions were excluded: obesity (body mass index ≥ 2) or history of prematurity. None of the included patients were taking medication or had an underlying condition that might affect the respiratory pattern during sleep.

### Polysomnography

Diagnosis of OSAS was based in a combination of clinical signs and symptoms and a full-night PSG. All children underwent nocturnal PSG at Pediatric Sleep Center at Professor Doutor Fernando Fonseca Hospital (level I PSG) and data were analyzed by the same department, according to American Academy of Sleep Medicine guidelines. The following variables were continuously measured and recorded by a computerized polysomnograph (Philips Respironics Alice): electroencephalography; electro-oculography; electromyography of anterior tibialis and chin muscles; electrocardiography; airflow was measured by means of nasal pressure cannula and thermistor; respiratory effort was measured by respiratory inductance plethysmography; transcutaneous oximetry and carbon dioxide monitoring; breathing noises; body position.

An apnea was considered when a drop of ≥ 90% in airflow compared with pre-event baseline respiration is seen. An apnea was classified as obstructive when it met apnea criteria for at least the duration of two breaths during baseline breathing and is associated with the presence of respiratory effort throughout the entire period of absent airflow. The obstructive apnea index (OAI) was defined as the number of obstructive apneas per hour of sleep. An apnea was classified as mixed when it met the apnea definition for at least the duration of two breaths during baseline breathing and was associated with absent respiratory effort during one portion of the event and the presence of inspiratory effort in another portion, regardless of which portion came first. The mixed apnea index (MAI) was defined as the number of mixed apneas per hour of sleep, and an MAI ≥ 1/h was considered abnormal. A central apnea was considered when it met apnea criteria and was associated with absent inspiratory effort throughout the entire duration of the event, and at least one of the following was met: the event lasted 20 s or longer, the event lasted at least the duration of two breaths during baseline breathing and was associated with an arousal or 3% or more oxygen desaturation. The central apnea index (CAI) was defined as the number of central apneas per hour of sleep, a CAI ≥ 1/h was considered abnormal and a central sleep apnea syndrome (CSAS) was defined when a CAI was superior to 5/h.

### Surgeries

All patients were submitted to AT. Adenoidectomy was performed with curettes. The technique used to perform tonsillectomy was extracapsular dissection with bipolar cautery.

### Statistical analysis

Statistical analysis was performed with SPSS version 26.0 software (International Business Machines Corporation, USA). Data were presented as median (25-75th percentile). Comparisons between groups were made by Wilcoxon Signed-Rank or Mann–Whitney *U* test, and correlations were made with Spearman’s Rank Correlation. Statistical significance was accepted at *p* < 0.05.

## Results

### Study population

Eighty child were eligible for inclusion in this study: 55 boys and 25 girls, with a median age of 3.6 years (2.1–5.9) years. Pre-operative median AHI was 14.1 (11.0–18.4) per hour, with a median preoperative OAI of 7.1 (4.1–10.6), MAI of 1.2 (0.6–1.6) and CAI of 1.0 (0.4–2.0). Tonsils were graded using the scheme by Brodsky et al. [13]: 2.5% of patients were classified as grade I, 16.2% as grade II, 51.3% as grade III, and 30.0% as grade IV. Tonsil grade and corresponding success rates are displayed in Table 1. There was no significant differences in success rate, preoperative MAI, OIA or CAI between subgroups of children accordingly with tonsil grades.

**Table 1:** Tonsil grade and corresponding success rates

Tonsil grade	Percentage of total	Success rate
Grade I	2.5	100
Grade II	16.2	76.9
Grade III	51.3	82.9
Grade IV	30.0	79.2

Tonsils were graded using the scheme by Brodsky et al. [13]

All 80 children were submitted to adenotonsillectomy as a treatment of OSAS. After surgery 42.5% of children had an OAI < 1/h, 46.3% an OIA between 1 and < 5/h, 3.8% an OIA ≥ 5/h. After adenotonsillectomy 2.6% of children had an AHI < 1/h, 78.6% and AHI between 1 and < 5/h and 18.8% of children had an AHI ≥ 5/h.

### Effect of adenotonsillectomy on mixed apneas

Adenotonsillectomy caused significant improvement in MAI, from 1.2 (0.6–1.6) to 0.5 (0.1–0.8) (*p* < 0.001). Preoperatively, a total of 61.3% of children had MAI ≥ 1/h, and 2.2% had an MAI > 5/h. After surgery 15.0% of children had an MAI ≥ 1/h. This represents a normalization of MAI in 91.7% of children that had an abnormal value prior surgery.

There was no significant correlation between MAI and AHI, OIA, CAI, mean and median oxygen saturation or age.

To investigate the effect of adenotonsillectomy on MAI, a group of 49 children with a MAI ≥ 1/h was selected. Characteristics of both groups are displayed in Table 2.

**Table 2:** Demographic and sleep parameters for subgroups of children with an MAI < 1/h and MAI ≥ 1/h at baseline

Variable	MAI < 1 *n* = 31	MAI ≥ 1 *n* = 49	*p* value
Male/female ratio	22/9	33/16	0.643
Age (years)	3.6 (2.1–5.9)	3.6 (3.2–6.9)	0.123
AHI (*n*/h)	13.5 (12.1–15.6)	16.3 (12.9–20.0)	0.065
OIA (*n*/h)	4.8 (2.9–8.3)	8.1 (5.6–11.5)	**0.027**
CAI (*n*/h)	0.7 (0.4–1.6)	1.1 (0.5–2.1)	0.156
Hypopnea index (*n*/h)	6.6 (3.5–8.6)	3.5 (1.9–5.6)	0.087
Mean Sat (%)	96.0 (94.0–97.0)	95.0 (93.3–97.0)	0.703
Min Sat (%)	90.0 (88.3–90.0)	90.0 (90.0–92.0)	0.234
ODI (*n*/h)	15.0 (8.8–23.5)	14.3 (9.9–24.3)	0.898
TST (min)	419.5 (408.8–478.0)	462.5 (422.0–472.3)	0.275
Stage 1 NREM (%)	0.3 (0.2–1.3)	0.8 (0.2–1.9)	0.165
Stage 2 NREM (%)	55.9 (48.7–61.5)	57.6 (52.9–61.7)	0.220
Stage 3 NREM (%)	30.6 (24.4–34.6)	27.9 (22.2–33.1)	0.424
REM sleep (%)	16.2 (11.5–18.8)	12.9 (8.1–16.5)	0.100

Data are presented as median (25-75th percentile). Wilcoxon Signed-Rank Test

*AHI* apnea hypopnea index, *CAI* central apnea index, *Min* minutes, *Mini* minimum, *n/h* number per hour, *OAI* obstructive apnea index, *Sat* saturation, *TST* total sleep time

Bold *p* value: statistical significance accepted

Children with an abnormal MAI had significant higher OAI, when compared with children with an MAI < 1/h.

### Effect of adenotonsillectomy on central apneas

Surgery promoted significant improvements in CAI from 1.0 (0.4–2.0) to 0.5 (0.1–0.9) (*p* < 0.001). Preoperatively, a total of 51.3% of children had CAI ≥ 1/h, and 5.0% superior to 5/h. After surgery only 23.8% of children had a CAI ≥ 1/h, and none had a CAI > 5/h. This represents a normalization of CAI in 75.6% of children that had an abnormal value prior surgery. Pre-operative and postoperative comparisons of sleep parameters are displayed in Table 3.

**Table 3:** Pre-operative and postoperative comparisons of sleep parameters

Variable	Pre-operative	Post-operative	*p* value
AHI (*n*/h)	14.1 (11.0–18.4)	2.9 (2.1–4.4)	**< 0.001**
OAI (*n*/h)	7.1 (4.1–10.6)	1.1 (0.6–2.1)	**< 0.001**
MAI (*n*/h)	1.2 (0.6–1.6)	0.5 (0.1–0.8)	**< 0.001**
CAI (*n*/h)	1.0 (0.4–2.0)	0.5 (0.1–0.9)	**< 0.001**
Hypopnea index (*n*/h)	3.4 (1.4–6.9)	4.0 (1.0–8.0)	**< 0.001**
ODI (*n*/h)	13.6 (7.6–20.9)	1.9 (1.0–4.2)	**< 0.001**
Min Sat (%)	90.0 (85.0–92.0)	92.0 (90.0–94.0)	**< 0.001**
Mean Sat (%)	95.0 (93.0–97.0)	97.0 (96.0–98.0)	**< 0.001**

*AHI* apnea hypopnea index, *CAI* central apnea index, *Mini* minimum, *n/h* number per hour, *OAI* obstructive apnea index, *Sat* saturation

Bold *p* value: statistical significance accepted

A significant correlation was found between CAI and mean oxygen saturation (*r* = 0.3, *p* = 0.012). There was no significant correlation between CAI and AHI, OIA, minimum oxygen saturation or age.

## Discussion

More than half of children otherwise healthy with OSAS also had abnormal MAI (61.3% of children) and CAI (51.3%) during sleep, and 5% had CSAS. The data show that abnormal values of nonobstructive apneas are common in children with OSAS.

The majority of children included in this study had tonsillar hypertrophy (grade III and IV). No significant differences were seen in success rates, preoperative MAI, OIA or CAI between subgroups of children accordingly with tonsil grades. Could be expected that an increased tonsil size would be related with a higher AHI. There are some factors reported in the literature that could explain why this is not always encountered. It is known that subjective tonsil size assessment have interrater reliability. It is difficult to get a clear visual assessment of the tonsil in children, as they usually are not cooperative with a throat exam. Tonsil size grading scale assesses the tonsil position relative to tonsillar pillars, and not necessarily the tonsil size [14]. Howard et al. suggest that intra-oral subjective size assessment of tonsil size do not closely correlate with pediatric preoperative AHI. The authors advocate that objective tonsil size, considering the three-dimensional size of the tonsils, is more useful to predict AHI [15].

Central apneas may be caused by physiologic or idiopathic mechanisms or may be secondary to other medical conditions [7]. It is believed that abnormal CAI values in children with OSAS are a relatively common finding, and probably secondary to post-arousal effects of obstructive apneas and hypopneas. In children with OSAS and abnormal CAI, a higher PaCO_2_ set point is stablished during sleep. Following an arousal occurs a drop in PaCO_2_, and this may cause a temporary cessation of respiratory drive, resulting in central apnea [7, 8]. Previous studies have found a prevalence of CAI ≥ 1/h in children with OSAS ranging from 15 to 64%, and this study found a prevalence within this range (51.3%) [7, 8, 16]. The clinical importance of an abnormal CAI in the context of OSAS is debated, even in children with CSAS. In the absence of OSAS, CSAS often indicates a neurologic abnormality, and the opposite is true for CSAS in context of OSAS. Abnormal CAI in the context of OSAS is likely to resolve after AT in the majority of children, even in cases of children with neurologic abnormalities. Therefore OSAS should be treated prior to additional workup for causes of persistent abnormal CAI following AT, namely central nervous system abnormalities [7].

Mixed apneas are rarely found in healthy children [2, 4, 17]. In this study, in a population of otherwise healthy children with OSAS, almost all children had one or more mixed apneas during sleep, and 61.3% of children had a MAI ≥ 1/h. Since mixed apneas are pathophysiologically considered to be part of OSAS, some studies do not differentiate obstructive from mixed events [10, 11, 18]. The mentioned may help to justify the lack of studies reporting the prevalence of mixed apneas in children with OSAS. Since the implication of mixed events in OSAS has not been well studied, their impact in this syndrome tends to be ignored. Yang et al. reported that MAI is found more frequently in patients with severe OSAS, proposing that mixed events was associated with higher loop gain and consequently with ventilatory control instability [1]. This may justify the high frequency of abnormal MAI found in this group of children with moderate-to-severe OSAS. Other studies suggest that patients with OSAS with mixed apneas have more breathing irregularities, suggesting that central respiratory control of this patients are different from the ones with pure obstructive events [19, 20].

Adenotonsillectomy is the first-line treatment for children with OSAS, as it causes significant improvement in sleep-disordered breathing (SDB) in the majority of children. Despite that, it is defended that some groups of children with predominantly nonobstructive events may benefit from other management options [11, 12]. Depending on the definition of surgical success and on the presence of additional comorbidities, previous studies report adenotonsillectomy success rates ranging from 23 to 90% of children with OSAS [12]. Considering success rate of a postoperative AHI < 5/h, this study found that 81.2% of children had a successful treatment, a value within the range described in the literature. In this study, after adenotonsillectomy, reductions were seen not only in OAI from 7.1 (4.1–10.6) to 1.1 (0.6–2.1) (*p* < 0.001), but also in MAI from 1.2 (0.6–1.6) to 0.5 (0.1–0.8) (*p* < 0.001) and CAI from 1.0 (0.4–2.0) to 0.5 (0.1–0.9) (*p* < 0.001).

Previous studies already report significant reductions in CAI following adenotonsillectomy, with a tendency for the resolution of CSAS [7, 8, 13]. In this study, after adenotonsillectomy only 23.8% of children had a CAI ≥ 1/h, and none had CSAS. This represents a normalization of CAI in 75.6% of children that had an abnormal value prior surgery. The major reason pointed for this results is that following resolution of OSAS, the arousals secondary to obstructive events resolves, which are the major stimulus for central apneas [7].

In this study, after surgery 15.0% of children had an MAI ≥ 1/h, compared with 61.3% of children prior adenotonsillectomy. This represents a normalization of MAI in 91.7% of children that had an abnormal value prior surgery. So far, mixed apnea is pathophysiologically considered to be part of obstructive events, and so, the decrease of mixed events should result from the resolution of the upper airway obstruction [1, 10, 11]. In this study, no correlation was found between MAI and AHI, OIA or CAI. Another cause pointed for the emergence of mixed apneas in patients with OSAS is the poor ventilatory control stability related with severe OSAS [7]. In this study, that included children with moderate-to-severe OSAS, none had severe OSAS after surgical treatment. The significative AHI decrease may have caused improvements in ventilatory control stability, which may ultimately lead to significant reductions in MAI.

There are some limitations in our study, some of them because of its retrospective nature. For example, some patients were excluded due to incomplete preoperative polysomnographic data. Another limitation was the homogenous population studied, and so, the results of this study are applicable only to a highly selected population. There is limited clinical interest in mixed events, because they tend to be considered obstructive, and so few data were published on about this subject [1, 11]. The results of our study offer some valuable insight into this topic. Future studies should include randomized prospective trials to confirm the findings.

## Conclusion

Non obstructive apneas are common in children with OSAS. Adenotonsillectomy lead to significant improvements in nonobstructive events, with a tendency to resolution of mixed and central apneas abnormalities. The present study provides additional support to the increasing debate about the possible slight clinical relevance of CAI in children with OSAS, before AT, especially if CAI ≤ 5.

## Data Availability

The data that support the findings of this study cannot be shared openly to protect study participant privacy and are available from the corresponding author upon reasonable request and with permission from the local ethics committee. Data are located in controlled access data storage at Hospital Professor Doutor Fernando Fonseca.
